# Secreted Frizzled-Related Protein-2 Inhibits Doxorubicin-Induced Apoptosis Mediated through the Akt-mTOR Pathway in Soleus Muscle

**DOI:** 10.1155/2018/6043064

**Published:** 2018-08-01

**Authors:** Hilda Merino, Dinender K. Singla

**Affiliations:** ^1^Department of Internal Medicine, College of Medicine, University of Central Florida, Orlando, FL, USA; ^2^Division of Metabolic and Cardiovascular Sciences, Burnett School of Biomedical Sciences, College of Medicine, University of Central Florida, Orlando, FL 32816, USA

## Abstract

Doxorubicin (Dox) is a potent chemotherapeutic drug known for its dose-dependent and serious adverse effects, such as cardiotoxicity and myotoxicity. Dox-induced cardiotoxicity (DIC) and muscle toxicity (DIMT) have been studied; however, the mechanisms of Dox-induced apoptosis in soleus muscle are not well defined. Our data shows that with Dox treatment, there is a significant increase in oxidative stress, apoptosis, proapoptotic protein BAX, pPTEN levels, and wnt3a and *β*-catenin activity (*p* < 0.05). Moreover, Dox treatment also resulted in decreased antioxidant levels, antiapoptotic BCL2, pAKT, p-mTOR, and endogenous levels of sFRP2 in the soleus muscle tissue (*p* < 0.05). Secreted frizzled-related protein 2 (sFRP2) treatment attenuated the adverse effects of DIMT and apoptosis in the soleus muscle, evidenced by a decrease in oxidative stress, apoptosis, BAX, pPTEN, and wnt3a and *β*-catenin activity, as well as an increase in antioxidants, BCL2, pAKT, p-MTOR, and sFRP2 levels (*p* < 0.05). This data suggests that Dox-induced oxidative stress and apoptosis is mediated through both the Akt-mTOR and wnt/*β*-catenin pathways. Moreover, the data also shows that sFRP2 modulates these two pathways by increasing signaling of Akt-mTOR and decreased signaling of the wnt/*β*-catenin pathway. Therefore, our data suggests that sFRP2 has valuable therapeutic potential in reversing Dox-induced oxidative stress and apoptosis in soleus muscle mediated through the Akt-mTOR pathway.

## 1. Introduction

Doxorubicin (Dox) is a well-known medication used to treat various types of cancer, including those related to the breast, lung, stomach, and blood [1]. Though it is effective as an antimalignancy agent, there are multiple serious side effects associated with its use, including damage to the heart (cardiotoxicity), skeletal tissue (myotoxicity), hair loss, and arrhythmia [2]. Therefore, the use of doxorubicin has been limited, and various alternative strategies have been planned.

Dox has been found to induce both acute and late-onset dysfunction of the heart, eventually leading to heart failure and potentially death [3, 4]. Dox-induced muscle toxicity (DIMT) can also cause dose-dependent muscle dysfunction; however, DIMT is associated with adverse changes to skeletal muscle tissue, leading to effects such as fatigue, atrophy, and eventually muscle cell death [5–8]. Significant loss in muscle can result in decreased response to treatment, worsening of prognosis, and a reduction in quality of life [5, 7]. Although the mechanism of Dox-induced cardiotoxicity has been studied extensively, the exact mechanism responsible for DIMT in soleus muscle has yet to be fully understood.

Dox treatment causes cardiac toxicity and involves multiple mechanisms, such as induced oxidative stress, inflammation, necrosis, apoptosis, and fibrosis [1, 2]. However, it remains unknown whether Dox toxicity in skeletal muscle involves these mechanisms or if it adopts a different pathway. Therefore, we proposed an investigation of oxidative stress-induced apoptosis in soleus muscle. Furthermore, these studies were extended to investigate mechanisms of apoptosis mediated by the Akt-mTOR pathway.

Moreover, the wnt/*β*-catenin pathway was traditionally viewed to serve a role in development [9]. However, the heart is now known to activate various pathways such as the wnt/*β*-catenin signaling pathway under states of stress [10]. During cardiac remodeling, inhibition of the wnt-signaling pathway at the soluble frizzled receptor level has been shown to be beneficial in repairing the damaged tissue [10]. This pathway has also been shown to have involvement in skeletal muscle remodeling [11]. A recent study showed a shift in skeletal muscle fiber type of both the quadriceps and soleus muscles in the dilated cardiomyopathy (DCM) mouse model, leading to skeletal myopathy [11]. Induction of tissue damage and repair also depends on specific activation of wnts. For example, wnt3a activation has been shown to induce apoptosis in the heart [12]. Therefore, we also designed the study to investigate if Dox-induced apoptosis of the skeletal muscle involves the wnt3a pathway.

Secreted frizzled-related proteins (sFRP) are considered to be antagonists of the wnt-signaling pathway [9, 13]. Therefore, these proteins can be used to inhibit the wnt signaling pathway and have been shown to be beneficial in disease states [12, 14–16]. sFRP2 is a part of the sFRP family and has been previously shown to reduce fibrosis and improve the left ventricular functionality of the heart in the rat myocardial infarction model [15]. Furthermore, sFRP2 has been shown to be a significant paracrine factor for stem cells, aiding in repair of the myocardium [16]. This suggests that if wnt signaling is involved in DIMT, this potentially could be a treatment option that is clinically significant in muscle tissue in addition to the heart.

The significant side effects of Dox give rise to an urgent need to understand the molecular mechanisms of the disease state in order to generate new treatment options and improve patients' quality of life. To the best of our knowledge, there are no studies on the role of sFRP2 in doxorubicin-induced toxicity of the soleus muscle mediated through the Akt-mTOR and wnt3a/*β*-catenin pathways. Therefore, this study was designed to investigate if oxidative stress-induced apoptosis is mediated through the Akt-mTOR and wnt3a/*β*-catenin pathways in the soleus muscle as well as to understand whether this process can be inhibited by sFRP2.

## 2. Materials and Methods

### 2.1. Study Groups

C57BL/6 mice were divided into three treatment groups: control (saline), Dox, and Dox + sFRP2, with *n* = 8 in each group. The Institutional Animal Care and Use Committee of the University of Central Florida approved the animal protocol used in this study.

### 2.2. Doxorubicin and sFRP2 Treatment

C57BL/6 male and female mice of eight to ten weeks of age were administered a dose of 4 mg/kg doxorubicin (Fisher Scientific, cat. number BP 2516-50) one time every other day (M, W, and F) via intraperitoneal (IP) injection, resulting in a cumulative dose of 12 mg/kg. Recombinant mouse sFRP2 (Sino Biological Inc., cat. number 50028-M08H) was reconstituted according to the manufacturer's instructions and injected via the tail vein at day one (D1) and day six (D6) after the final Dox injection at a dose of 40 *μ*g/kg, for a cumulative dose of 80 *μ*g/kg.

### 2.3. Tissue Harvest and Paraffinization

Mice were sacrificed, and bilateral soleus muscle was harvested at day 14 (D14). The left soleus was kept in RNA later, and the right soleus was kept in 10% paraformaldehyde (PFA) for storage. Paraffin blocks were made of the samples, and blocks were sectioned (5 *μ*m) and placed on ColorFrost Plus microscope slides (Fisher Scientific, cat. number 12-550-17).

### 2.4. Catalase, MnSOD, and Lipid Peroxide Assays

Catalase activity was measured as previously reported [3] using a colorimetric assay kit (Abcam, cat. number ab83464). The colorimetric assay was measured at 570 nm, and these were adjusted for total protein concentration.

Manganese-containing mitochondrial superoxide dismutase (MnSOD) levels were analyzed as previously reported [3] using an assay kit (Applied Bioanalytical Labs, cat. number SOD-560). The manufacturer's protocol was followed, and the absorbance of the sample was measured at 560 nm, using a plate reader.

Lipid peroxides were analyzed following the LPO-CC assay kit per the manufacturer's instructions (Kamiya Biomedical Co., cat. number CC-004) and as previously reported by this lab [3]. This sample was analyzed at 675 nm with a Bio-Rad plate reader.

### 2.5. Dihydroethidium Staining

Dihydroethidium (DHE) (Invitrogen cat. number D23107) staining was performed as previously reported [17]. The samples were deparaffinized and incubated with DHE (1 *μ*m/mL) for 15–25 minutes at room temperature in the dark. The samples were then washed with phosphate-buffered saline (PBS) and counterstained with DAPI in order to determine total nuclei count. Confocal microscopy was used for representative imaging.

### 2.6. Myosin Staining

Myosin staining was performed to show the soleus muscle tissue. Before staining, the sections were deparaffinized and rehydrated using sequential decreasing alcohol concentration. They were then blocked in 10% normal goat serum (NGS) for one hour and decanted thereafter. Antimyosin (raised in rabbit, Sigma-Aldrich, cat. number M7523) was added in 10% NGS at a 1 : 50 concentration. This was incubated overnight at 4°C. The sections were then washed with 1x PBS, and Alexa Fluor 488 goat anti-rabbit (Invitrogen, cat. number A11008) was added in 1x PBS for 1 hour at room temperature. Finally, the sections were washed with 1x PBS.

### 2.7. TUNEL Staining

TUNEL staining was performed to determine the percentage of apoptotic nuclei (TMR Red, Roche, cat. number 12156792910). Slides prepared with soleus muscle tissue sections were deparaffinized and permeabilized with proteinase K (25 *μ*g/mL in 100 mM Tris-HCl), as previously described [17]. The sections were counterstained and mounted with Antifade Mounting Medium with 4′,6-diamidino-2-phenylindole (DAPI) (Vector Laboratories, cat. number H-1200) to show the total nuclei. The percentage of apoptotic cells was quantified by dividing the number of TUNEL-positive cells by the total nuclei. Confocal microscopy was used to obtain representative images of the muscle tissue.

### 2.8. Caspase-3 Immunohistochemistry and ELISA

Caspase-3 staining utilizing anti-caspase-3 (Rabbit, Abcam, cat. number ab13847) was performed to corroborate apoptosis' involvement in DIMT. This stain was carried out following standard staining procedures, as published previously [2]. In brief, the samples were blocked with NGS and washed with 1x PBS, and the primary antibody, anti-caspase-3 (1 : 50), was diluted in 10% NGS and incubated at 4°C overnight. The slides were washed with 1x PBS, and the secondary antibody, Alexa Fluor 568 goat anti-rabbit, was added and allowed to incubate for one hour at room temperature. The slides were washed one more time and finally were mounted and stained with DAPI.

Caspase-3 activity was analyzed using an ELISA kit from BioVision according to the manufacturer's instructions and as previously reported [17]. Soleus muscle was washed with 1x PBS and homogenized in cell lysis buffer. The sample was then centrifuged to isolate the supernatant. Then, the sample was analyzed to determine protein concentration at 405 nm using a plate reader.

### 2.9. pPTEN, pAKT, Wnt3A, and *β*-Catenin ELISA Analyses

pPTEN was analyzed using the PathScan Phospho-PTEN (Ser380) Sandwich ELISA kit (Cell Signaling Technology, cat. number 7285), as previously reported [3]. In brief, the soleus muscle tissue was homogenized. Then, protein concentration was estimated using the Bradford Assay, and the tissue was incubated for 2 hours at 37°C. Next, a colorimetric assay was performed following the manufacturer's instructions. Once complete, stop solution was added, and the samples were read at 450 nm using the ELISA plate reader. Data was expressed in arbitrary units (AU).

pAKT activity was measured using a commercially available kit (Exalpha Biologicals Inc., cat. number X1844K), as previously reported [3]. In brief, soleus muscle tissue was homogenized, and protein estimation was performed. The sample was incubated for 2 hours, and a colorimetric assay was performed per the manufacturer's instructions. Using a Bio-Rad plate reader, samples were analyzed at 450 nm. Data was expressed in AU.

A WNT3A ELISA kit (USCN Life Sciences Inc., cat. number E83155Hu) was used to determine WNT3A activity per the manufacturer's instructions. In brief, the kit's detection reagent A was added to the samples and they were incubated at 37°C for 1 hour. The solution was washed, and then detection reagent B was added and incubated for another 30 minutes at 37°C. The samples were washed again, and the substrate solution was added to the samples and incubated for 10–15 minutes at 37°C. Finally, stop solution was added, and the samples were analyzed at 450 nm using a plate reader.

*β*-Catenin was analyzed using a commercially available ELISA kit (Enzo Life Sciences, cat. number ADI-900-135) following the manufacturer's instructions. In brief, the samples were added to the assay buffer and allowed to sit at room temperature for 1 hour on a shaker. The primary antibody was added to each well and incubated for another hour on the shaker. The plate was washed 5 times, and 100 *μ*L of blue conjugate was added. The plate was washed 5 more times, and 100 *μ*L of soluble substrate was added and incubated for another 30 minutes at room temperature on the shaker. Finally, stop solution was added, and the samples were analyzed using a plate reader at 450 nm.

### 2.10. Western Blot Analysis of p-mTOR, sFRP2, BCL2, and BAX

sFRP2 presence was confirmed with Western blot analysis, using a standard Western blotting procedure. In brief, sFRP2 antibody (Abcam, cat. number ab111874) at a 1 : 250 concentration was analyzed with *β*-actin (1 : 1000) as a loading control. The secondary antibody was anti-rabbit at a concentration of 1 : 1000 for both.

Analysis of p-mTOR over total mTOR was performed using Western blot, following standard technique. Primary antibodies, p-mTOR and mTOR, were used at concentrations 1 : 1000 and 1 : 750, respectively (Cell Signaling, cat. number 2971L and 2972S). The secondary antibody for both was HRP-conjugated anti-rabbit, at concentrations of 1 : 1000 and 1 : 750, respectively.

Western blot analysis was performed to analyze BCL2, an antiapoptotic protein, and BAX, a proapoptotic protein, following a standard Western blotting procedure (Cell Signaling Technology, cat. numbers SC-492 and 2772, resp.). The primary antibodies were BCL2 and BAX, both at a 1 : 100 concentration. The secondary antibody was HRP-conjugated anti-rabbit for both BCL2 and BAX, at a concentration of 1 : 1000. Quantitative densitometry analysis of Western blotting was performed using ImageJ software (NIH).

### 2.11. Statistical Analysis

The data is expressed as mean ± SE. Statistical significance was determined when *p* < 0.05, using one-way ANOVA and Tukey's test.

## 3. Results

### 3.1. Effects of sFRP2 on Oxidative Stress (Lipid Peroxidases) and Antioxidants (MnSOD and Catalase)

**Figure 1:**
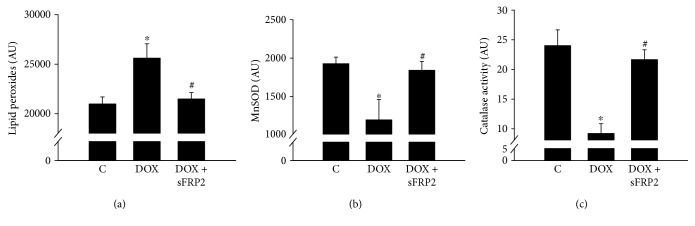
Effect of sFRP2 treatment on lipid peroxides, superoxide dismutase, and catalase activity.  Figure 1 shows quantitative data from the ELISA kits for lipid peroxides (a) to determine oxidative injury to the muscle, MnSOD (b) to determine the presence of the antioxidant superoxide dismutase, and (c) to determine the presence of the antioxidant, catalase. Units represented in arbitrary units. ^∗^*p* < 0.05 compared to control, and ^#^*p* < 0.05 compared to the Dox group. *n* = 4-5 for lipid peroxides, *n* = 5-6 for MnSOD, and *n* = 6 for catalase activity.

Figure 1(a) shows quantitative ELISA analysis of an oxidative stress marker, lipid peroxidase. Dox treatment shows a significant increase of lipid peroxidases; however, this increase was significantly decreased by sFRP2 treatment (Figure 1(a), *p* < 0.05). Furthermore, we performed ELISAs to detect the levels of antioxidants, MnSOD and catalase. Following Dox treatment, there was a decrease in antioxidants significantly, whereas sFRP2 treatment significantly increased MnSOD and catalase (Figures 1(b) and 1(c), *p* < 0.05). This data suggests that sFRP2 treatment improves antioxidant levels in Dox-treated soleus muscle (Figures 1(b) and 1(c), *p* < 0.05).

### 3.2. Effects of sFRP2 Treatment on Oxidative Stress Marker DHE

**Figure 2:**
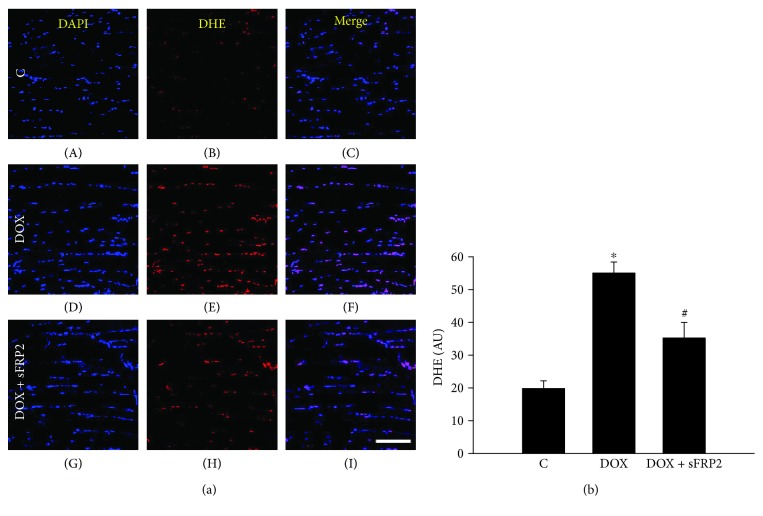
Significant decrease in DHE-positive cells post-sFRP2 treatment. (a) shows DAPI staining to determine the total number of nuclei in (A, D, and G), DHE staining to measure oxidative stress levels in (B, E, and H), and the merged photomicrographs (C, F, and I). (b) shows the quantitative immunohistochemistry data for the DHE staining. Units represented in arbitrary units. ^∗^*p* < 0.05 compared to control, and ^#^*p* < 0.05 compared to the Dox group. Scale for A is 100 *μ*m. *n* = 4-5.

Figure 2(a) shows staining for total nuclei in blue with DAPI (A, D, and G), DHE stain in red to determine superoxide levels (B, E, and H), and the merged images (C, F, and I). Quantitative analysis of DHE-positive cells shows that with treatment of Dox, superoxide levels significantly increased (Figure 2(b), *p* < 0.05). This significant increase was attenuated with sFRP2 treatment, further suggesting that sFRP2 treatment inhibits increased oxidative stress (Figure 2(b), *p* < 0.05), in a similar fashion observed with lipid peroxidase in Figure 1(a).

### 3.3. Effects of sFRP2 on Apoptosis and Caspase-3 Activity

**Figure 3:**
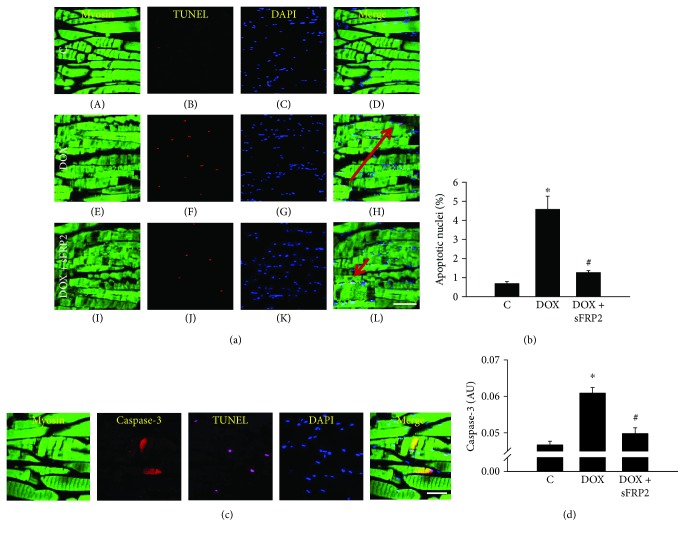
sFRP2 treatment decreases caspase-3 activity and inhibits apoptosis. (a) shows representative imaging of soleus muscle. The muscle has been stained with antimyosin (A, E and I), TUNEL to confirm apoptosis (B, F, and J), and DAPI to determine total nuclei (C, G, and K), and the merged images of all staining can be seen (with enlargements, denoted by a red arrow) in (D, H, and L). (b) shows a graph of the quantitative data from immunohistochemistry for the percentage of apoptotic nuclei. (c) shows a stain of the soleus muscle using antimyosin, caspase-3, TUNEL, and DAPI, from left to right. (d) shows the quantitative results from an ELISA kit for caspase-3 activity, a key mediator in apoptosis. Units represented in percentage of apoptotic nuclei in (b) and in arbitrary units for caspase-3 activity in (d). ^∗^*p* < 0.05 compared to control, and ^#^*p* < 0.05 compared to the Dox group. Scale for (a) is 100 *μ*m. Scale for (c) is 50 *μ*m. *n* = 5-6 for apoptotic nucleus percentage, and *n* = 7-8 for caspase-3 activity.

Figure 3(a) shows detection of apoptosis by TUNEL staining. The muscle tissue is stained for myosin in green in A, E, and I; the apoptotic nuclei are stained in red as seen in B, F, and J; total nuclei are stained in C, G, and K; and the merged images are seen in D, H, and L (Figure 3(a)).

Quantitative analysis of the apoptotic nuclei was obtained.  Figure 3(b) shows a graph of the percentage of apoptotic nuclei. Our data shows a significant increase in the number of apoptotic nuclei in the Dox treatment group compared to the control; however, this increase was significantly decreased following treatment with sFRP2 (*p* < 0.05).

An additional staining was performed to determine the presence of caspase-3 in apoptotic muscle, as seen in Figure 3(c). From left to right, myosin, caspase-3, TUNEL, DAPI, and merged images show that the TUNEL-positive soleus muscle is also positive with caspase-3, suggesting that Dox-induced apoptosis does occur in the soleus muscle. Moreover, we performed a caspase-3 ELISA to quantify apoptosis in these soleus muscle cells (Figure 3(d)). Figure 3(d) shows a significant increase in caspase-3 activity following treatment with Dox; however, this increase in caspase-3 activity was attenuated with sFRP2 treatment (*p* < 0.05). Noticeably, our TUNEL staining corresponds with the additional method of caspase-3 activity ELISA analysis, suggesting that apoptosis is occurring in soleus muscle and that this apoptosis is attenuated by sFRP2.

### 3.4. Effects of sFRP2 on Proapoptotic Protein BAX and Antiapoptotic BCL2

**Figure 4:**
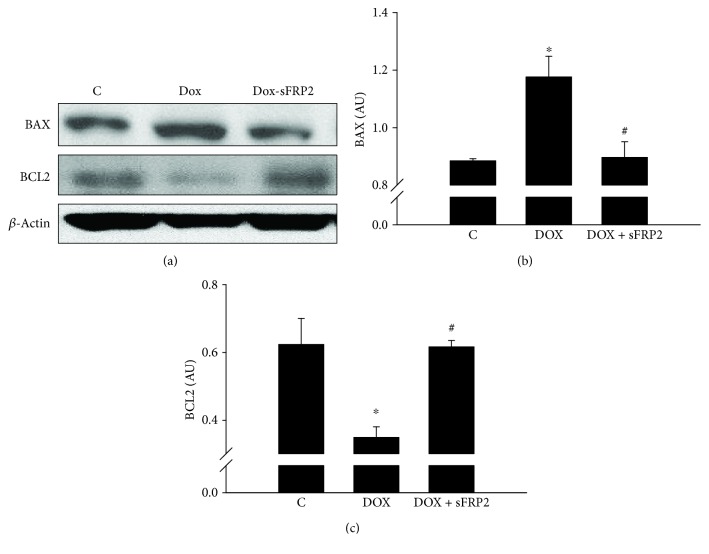
sFRP2 treatment post-administration of Dox decreases proapoptotic protein BAX and increases antiapoptotic protein BCL2.  Figure 4 shows a Western blot analysis of BAX (proapoptotic), BCL2 (antiapoptotic), and *β*-actin as the control. Qualitative data can be seen in (a), and quantitative data in the form of graphs can be seen below for BAX in (b) and BCL2 in (c). Units represented in arbitrary units. ^∗^*p* < 0.05 compared to control, and ^#^*p* < 0.05 compared to the Dox group. *n* = 3 for both BAX and BCL2.

Figure 4(a) shows the Western blot on BAX and BCL2, with *β*-actin as the loading control. The quantitative Western blot analysis shows that Dox induces an increase in proapoptotic protein BAX and a decrease in antiapoptotic protein BCL2 (*p* < 0.05). The BAX levels significantly decreased after sFRP2 treatment, and BCL2 level significantly increased compared to the Dox group (Figures 4(b) and 4(c), resp., *p* < 0.05), suggesting that sFRP2 inhibits apoptosis in the soleus muscle induced by Dox.

### 3.5. sFRP2 Treatment on PTEN, AKT, and mTOR

**Figure 5:**
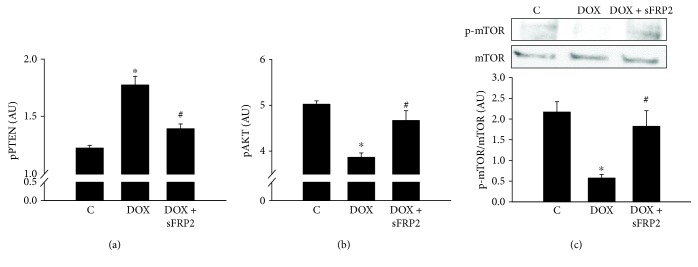
sFRP2 treatment decreases pPTEN levels and increases pAKT and p-mTOR/mTOR. Figure 5 shows the quantitative results of the ELISA kits for pPTEN (a) and pAKT (b). (c) shows analysis of p-mTOR over total mTOR activity using Western blot. Units represented in arbitrary units. ^∗^*p* < 0.05 compared to control, and ^#^*p* < 0.05 compared to the Dox group. *n* = 7 for pPTEN and *n* = 6-7 for pAKT. *n* = 4-5 for p-mTOR/mTOR analysis.

Figure 5 shows the quantitative data for ELISAs performed on pPTEN and pAKT, whereas Western blot analysis was performed to detect p-mTOR. When treated with Dox, the pPTEN levels significantly increased; however, this increase was mitigated by the addition of sFRP2 (Figure 5(a), *p* < 0.05). In contrast, when treated with Dox, the pAKT levels significantly decreased, and this decrease was then attenuated by the addition of sFRP2 (Figure 5(b), *p* < 0.05). The Western blot in Figure 5(c) shows a significant decrease in p-mTOR, whereas total mTOR was used as a loading control. Densitometry quantification shows a significant decrease in p-mTOR with Dox treatment; however, this decrease in p-mTOR was significantly increased following sFRP2 treatment (*p* < 0.05). This data suggests involvement of the Akt-mTOR pathway in Dox-induced apoptosis in the soleus muscle.

### 3.6. Effects of sFRP2 Treatment on Wnt3a and *β*-Catenin

**Figure 6:**
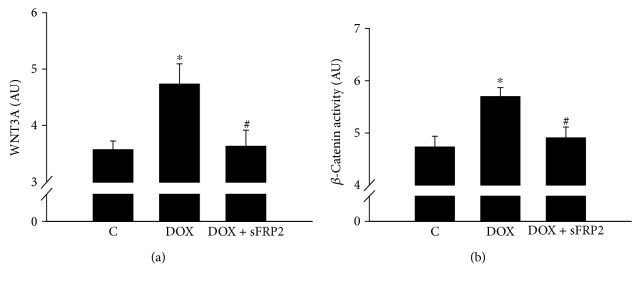
Wnt3a and *β*-catenin decrease with sFRP2 administration. Figure 6 shows graphs for the quantitative data from the ELISA assay kits for wnt3a to determine the involvement of the wnt signaling pathway and *β*-catenin to determine levels of this signaling molecule (a and b, resp.). Units represented in arbitrary units. ^∗^*p* < 0.05 compared to control, and ^#^*p* < 0.05 compared to the Dox group. *n* = 6-7 for wnt3a, and *n* = 6 for *β*-catenin activity.

Figure 6 shows quantitative ELISA analysis of wnt3a and *β*-catenin. Our data shows a significant increase in both wnt3a and *β*-catenin with Dox treatment; however, this increase was reduced with sFRP2 treatment (Figures 6(a) and 6(b), resp., *p* < 0.05). This is indicative of the wnt3a/*β*-catenin pathway's involvement in Dox-induced cytotoxicity of the soleus muscle and that sFRP2 attenuates the wnt3a/*β*-catenin pathway.

### 3.7. Determining Levels of sFRP2 in Soleus Muscle

**Figure 7:**
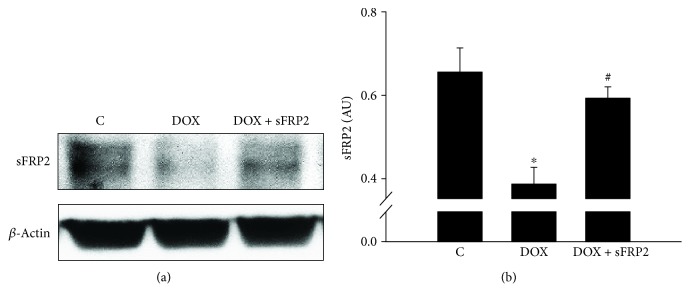
Endogenous presence of sFRP2 in soleus muscle. (a) shows a qualitative Western blot of sFPR2 with *β*-actin as the loading control. To the right of this Western blot picture in (b) is the quantitative results from the sFRP2 densitometry analysis using ImageJ software (NIH), confirming its presence in the soleus muscle. Units represented in arbitrary units. ^∗^*p* < 0.05 compared to control, and ^#^*p* < 0.05 compared to the Dox group. *n* = 4-5 for sFRP2.

Figure 7(a) shows Western blot analysis of sFRP2's presence in the soleus muscle tissue, with and without Dox treatment, with *β*-actin as the loading control. Our data shows a decrease in sFRP2 in the Dox treatment group compared to the control (Figure 7(b), *p* < 0.05). This decrease in sFRP2 was attenuated following administration of sFRP2 (Figure 7(b), *p* < 0.05).

We have also developed a flow chart to demonstrate that the wnt3a and Akt-mTOR pathways are involved in Dox-induced apoptosis of the soleus muscle.

## 4. Discussion

Doxorubicin is a chemotherapeutic drug known to induce cardiotoxicity and myotoxicity as major side effects [2, 18, 19]. A previous study has reported that when treated with Dox, the skeletal muscle gives rise to increased muscle fatigue and reduced blood flow, interferes with actin-myosin interaction and contractile alterations, and results in overall lower functionality [19]. The aforementioned adverse effects warrant study for potential therapeutics to attenuate muscle toxicity induced by doxorubicin. The major new and important information in the present work, following Dox toxicity in the soleus muscle and protective effects of sFRP2 treatment, includes (1) a decrease in oxidative stress markers, lipid peroxide, and DHE; (2) an increase in antioxidant levels of MnSOD and catalase; (3) a decrease in soleus muscle cell apoptosis; (4) a decrease in proapoptotic protein BAX and an increase in antiapoptotic protein BCL2; (5) a decrease in the negative regulation of PTEN and an increase in cell survival proteins pAKT and p-mTOR; (6) effects on the wnt3a/*β*-catenin pathway by decreasing wnt3a and *β*-catenin activity; (7) and, finally, an improvement of the decrease seen in endogenous sFPR2 in Dox-treated animals. The findings that sFRP2 inhibits oxidative stress and improves antioxidant levels in the soleus muscle are consistent with other published studies on decreased oxidative stress and apoptosis in skeletal muscle following exercise, as reported by Smuder et al. [20, 21]. Moreover, the role of increased oxidative stress and decreased antioxidant reserve following muscle injury in Duchenne muscular dystrophy (DMD) has also been reported, which is in agreement on the alteration of oxidative and antioxidant defense following Dox toxicity in muscle [22–25]. It has been shown in muscle atrophy and muscle inactivity studies that there is an increase in reactive oxygen species (ROS) and a decrease in antioxidants [26–29]. In contrast, recent data in a denervation study shows that antioxidant genes increase immediately as a result of increased ROS, suggesting that muscular atrophy and weakness is independent of the oxidative stress pathway [28]. Moreover, they further confirmed that the process is not mediated through oxidative stress, as antioxidants trolox and resveratrol were shown not to have an effect on oxidative stress-induced atrophy and muscle weakness [28]. Our data differed with this denervation study with respect to antioxidants and lipid peroxidase determination. For example, in their study, lipid peroxidase levels were not assessed related to oxidative stress and antioxidant levels were not changed [28], whereas our data in the DIMT model shows a significant increase in lipid peroxide levels as well as a decrease in the antioxidants in the Dox-treated group. Therefore, it is anticipated that the role of oxidative stress in muscle injury is mediated through two independent pathways: one mediated through increased oxidative stress and decreased antioxidant reserves as reported in the current study, and another through nonoxidative stress in the denervation study, as published previously [28]. Overall, our data is also consistent with Dox-induced cardiotoxicity and in various other muscle conditions such as atrophy that involve changes in oxidative stress and antioxidants [2–4, 15, 16, 18, 27, 30]. Moreover, we suggest that sFRP2, which has never been reported before, could be a potential target to decreasing oxidative stress and increasing antioxidant reserves following Dox treatment.

Apoptosis is a programmed cell death mediated by mitochondrial proteins, caspase-3 and -9, which has been reported in various skeletal muscle diseases such as exercise-induced muscle damage, dystrophinopathies, inflammatory myopathies, ischemic atrophy, and spinal muscular atrophy [31–36]. The presence of apoptosis is confirmed by TUNEL, BAX, and BCL2 staining, and this apoptosis significantly contributes to the development and progression of these disease states [32]. Moreover, a recent study shows that Dox induces apoptosis in rat soleus muscle [20, 21]. Data from another recent study shows that TNF-*α*-induced apoptosis in C2C12 myoblast cells involves a decrease in the BCL2 to BAX ratio, which is consistent with our data [37]. The current study corroborates the previously published muscle studies on Dox-induced muscle apoptosis. The presence of apoptosis in the current study is also confirmed by TUNEL staining, caspase-3 activity, proapoptotic BAX, and antiapoptotic BCL2, which is in agreement with other studies published on muscle apoptosis [32]. Moreover, the presence of apoptosis in muscle cells was further confirmed for caspase-3 and TUNEL occurring in single muscle cells, as confirmed with muscle-specific protein, myosin, and nuclear stain, DAPI (Figure 3). Therefore, this data confirms that apoptosis is present in injured soleus muscle cells following Dox treatment.

Next, we confirmed whether apoptosis in the soleus muscle is mediated through the Akt-mTOR pathway and/or the wnt3a/*β*-catenin pathway. The Akt-mTOR pathway plays a major role in cell processes such as cell proliferation, survival, growth, and death [3, 16, 30, 38, 39]. An *in vivo* study published in Nature Cell Biology suggests that the Akt-mTOR pathway plays a major role in skeletal muscle hypertrophy, where an increase in activity of this pathway resulted in decreased muscle atrophy [38]. Additionally, the role of Akt-mTOR pathway activation has also been shown to play a role in decreasing DMD-associated fibrosis and inflammation [39]. Additionally, a previous study has shown that the PI3K/Akt/mTOR pathway is involved in Dox-induced apoptosis in the heart and the process is inhibited by transplantation of embryonic stem cells [3]. However, the role of the Akt-mTOR pathway in Dox-induced apoptosis in soleus muscle is not well defined. Therefore, in the present study, as per the best of our knowledge, we are the first to report that pAKT and p-mTOR decrease significantly compared to the control following Dox treatment, whereas sFRP2 treatment significantly (*p* < 0.05) increases Akt and mTOR, suggesting involvement of the Akt-mTOR pathway in DIMT of the soleus muscle.

PTEN, an endogenous inhibitor of the Akt pathway, has been shown to be modulated in different muscle and cardiac diseases [3, 40]. A significant increase in PTEN and apoptosis was observed in insulin-resistant skeletal muscle cells following insulin stimulation [40]. This increase in PTEN was suppressed when cells were treated with metformin, a common drug for diabetes, suggesting that PTEN regulates apoptosis in this insulin-resistant muscle model [40]. In agreement with these studies, PTEN in the current study was significantly increased (*p* < 0.05) in Dox-treated mice along with apoptosis, whereas PTEN and apoptotic levels were significantly attenuated following administration of sFRP2 (*p* < 0.05). This data suggests that the PTEN pathway is involved in regulation of apoptosis mediated through the Akt-mTOR pathway in soleus muscle.

The wnt family consists of 19 members with discrete types of cellular functions, such as stem cell differentiation, myogenesis, cell survival, muscle fibrosis, and apoptosis, in organ development [41–44]. The function of wnts depends on their isoform, type of injury, and organ development [44]. Wnt3a treatment increases deposition of connective tissue in the muscle [43, 44]. Moreover, mouse embryos that were deficient in wnt1 and wnt3a demonstrate abnormal growth and a reduction in expression of muscle protein, Myf5 [44, 45]. Additionally, wnt3a has been published in previous studies of myocardial infarction-induced apoptosis, where increased levels of wnt3a were observed after the infarct was generated, whereas sFRP2 administration attenuated this wnt3a activity [15]. Noticeably, there is no published report on wnt3a and sFRP2 in DIMT. Therefore, our data on the increase in apoptosis as well as wnt3a and *β*-catenin activity is in agreement with the published myocardial infarction model and provides novel information in Dox-induced muscle toxicity. Moreover, the current study shows that an increase in wnt activity was significantly attenuated following treatment with sFPR2, which is also in agreement with studies published in the heart, and in some forms of cancer such as medulloblastoma [15, 46].

Next, we examined whether Dox treatment decreases exogenous levels of sFRP2 in soleus muscle, which may play a role in the increased oxidative stress and apoptosis seen in DIMT. Our data in Figure 7 shows that Dox treatment significantly decreases sFRP2 in soleus muscle, whereas treatment with sFRP2 injection brings back levels of this protein close to control values (*p* < 0.05). This suggests that baseline levels of sFRP2 are present in healthy skeletal muscle and play a protective role against various muscle disorders.

There are published studies that show significant decrease in muscle mass in early and late stages of cancer progression that ultimately leads to a decrease in muscle function [47–50]. It has been observed that this process can be mediated through inflammation from presence of a tumor [47, 48]. Interestingly, a recent study by Yu et al. report that the compound ghrelin was shown to inhibit Dox-induced apoptosis in the gastrocnemius muscle, suggesting a therapeutic role in the associated cancer cachexia [51]. Noticeably, we are suggesting in this study that sFRP2 inhibits apoptosis in soleus muscle, which could be a part of induced muscle cachexia as Yu et al. also report apoptosis in the gastrocnemius muscle. Based on our data on the decrease of apoptosis in Dox-induced muscle cachexia, we anticipate that sFRP2 could be a potential therapeutic target for cancer cachexia that is formed due to tumor formation.

**Figure 8:**
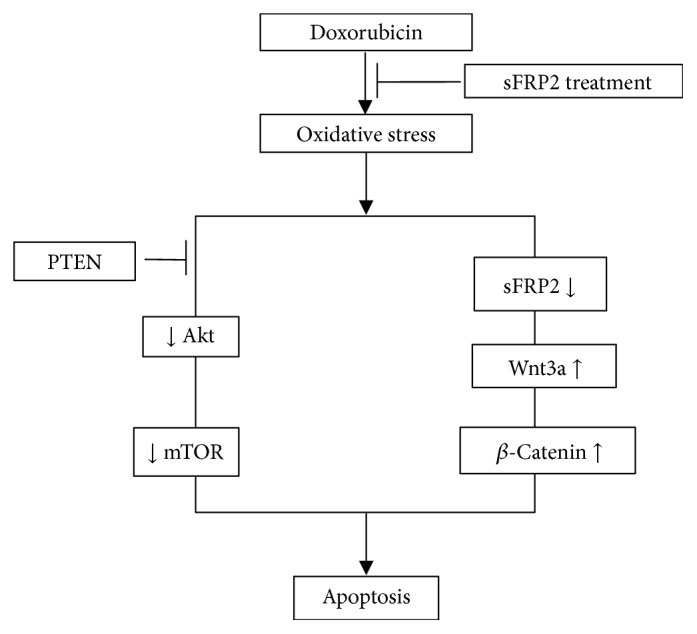
sFRP2 treatment inhibits oxidative stress induced by doxorubicin. Figure 8 is a flow chart that describes the effects of sFRP2 treatment on the two pathways involved in doxorubicin-induced apoptosis: Akt/mTOR and Wnt/*β*-catenin.

In conclusion, our data suggests that Dox induces oxidative stress and apoptosis and that the process is mediated through the Akt-mTOR and wnt3a/*β*-catenin pathways. We also presented a flow chart in Figure 8 to depict involvement of two pathways in Dox-induced muscle toxicity and apoptosis: Akt-mTOR and wnt3a/*β*-catenin. Interestingly, the link of wnt7a binding to frizzled protein-7 that activates the PI3K-Akt-mTOR pathway has been reported [44, 52]. However, in the current study we do not provide a link between wnt3a and the Akt-mTOR pathway in the regulation of apoptosis, which we propose as a future study by us or others. Finally, further studies in large animals are needed to confirm these findings, so that sFRP2 can be potentially used in the clinical setting.

## Data Availability

The data generated in the present study is available upon request.

## References

[1] Singal P. K., Iliskovic N. (1998). Doxorubicin-induced cardiomyopathy. *The New England Journal of Medicine*.

[2] Singla D. K., Ahmed A., Singla R., Yan B. (2012). Embryonic stem cells improve cardiac function in doxorubicin-induced cardiomyopathy mediated through multiple mechanisms. *Cell Transplantation*.

[3] Singla D. K. (2015). Akt-mTOR pathway inhibits apoptosis and fibrosis in doxorubicin-induced cardiotoxicity following embryonic stem cell transplantation. *Cell Transplantation*.

[4] Singla D. K., Abdelli L. S. (2015). Embryonic stem cells and released factors stimulate c-kit^+^/FLK-1^+^ progenitor cells and promote neovascularization in doxorubicin-induced cardiomyopathy. *Cell Transplantation*.

[5] Argiles J. M., Busquets S., Stemmler B., Lopez-Soriano F. J. (2014). Cancer cachexia: understanding the molecular basis. *Nature Reviews Cancer*.

[6] Gouspillou G., Scheede-Bergdahl C., Spendiff S. (2015). Anthracycline-containing chemotherapy causes long-term impairment of mitochondrial respiration and increased reactive oxygen species release in skeletal muscle. *Scientific Reports*.

[7] Nissinen T. A., Degerman J., Rasanen M. (2016). Systemic blockade of ACVR2B ligands prevents chemotherapy-induced muscle wasting by restoring muscle protein synthesis without affecting oxidative capacity or atrogenes. *Scientific Reports*.

[8] Tavakoli Dargani Z., Singla R., Johnson T., Kukreja R., Singla D. K. (2018). Exosomes derived from embryonic stem cells inhibit doxorubicin and inflammation-induced pyroptosis in muscle cells. *Canadian Journal of Physiology and Pharmacology*.

[9] Clevers H., Nusse R. (2012). Wnt/*β*-catenin signaling and disease. *Cell*.

[10] Bergmann M. W. (2010). WNT signaling in adult cardiac hypertrophy and remodeling: lessons learned from cardiac development. *Circulation Research*.

[11] Okada K., Naito A. T., Higo T. (2015). Wnt/*β*-catenin signaling contributes to skeletal myopathy in heart failure via direct interaction with forkhead box O. *Circulation: Heart Failure*.

[12] Zhang Z., Deb A., Zhang Z. (2009). Secreted frizzled related protein 2 protects cells from apoptosis by blocking the effect of canonical Wnt3a. *Journal of Molecular and Cellular Cardiology*.

[13] Niehrs C. (2012). The complex world of WNT receptor signalling. *Nature Reviews Molecular Cell Biology*.

[14] Alfaro M. P., Vincent A., Saraswati S. (2010). sFRP2 suppression of bone morphogenic protein (BMP) and Wnt signaling mediates mesenchymal stem cell (MSC) self-renewal promoting engraftment and myocardial repair. *Journal of Biological Chemistry*.

[15] He W., Zhang L., Ni A. (2010). Exogenously administered secreted frizzled related protein 2 (Sfrp2) reduces fibrosis and improves cardiac function in a rat model of myocardial infarction. *Proceedings of the National Academy of Sciences of the United States of America*.

[16] Mirotsou M., Zhang Z., Deb A. (2007). Secreted frizzled related protein 2 (Sfrp2) is the key Akt-mesenchymal stem cell-released paracrine factor mediating myocardial survival and repair. *Proceedings of the National Academy of Sciences of the United States of America*.

[17] Fatma S., Selby D. E., Singla R. D., Singla D. K. (2010). Factors released from embryonic stem cells stimulate c-kit-FLK-1^+ve^ progenitor cells and enhance neovascularization. *Antioxidants & Redox Signaling*.

[18] Zhao L., Zhang B. (2017). Doxorubicin induces cardiotoxicity through upregulation of death receptors mediated apoptosis in cardiomyocytes. *Scientific Reports*.

[19] Hydock D. S., Lien C. Y., Jensen B. T., Schneider C. M., Hayward R. (2011). Characterization of the effect of in vivo doxorubicin treatment on skeletal muscle function in the rat. *Anticancer Research*.

[20] Smuder A. J., Kavazis A. N., Min K., Powers S. K. (2011). Exercise protects against doxorubicin-induced markers of autophagy signaling in skeletal muscle. *Journal of Applied Physiology*.

[21] Smuder A. J., Kavazis A. N., Min K., Powers S. K. (2011). Exercise protects against doxorubicin-induced oxidative stress and proteolysis in skeletal muscle. *Journal of Applied Physiology*.

[22] Kim J. H., Lawler J. M. (2012). Amplification of proinflammatory phenotype, damage, and weakness by oxidative stress in the diaphragm muscle of *mdx* mice. *Free Radical Biology & Medicine*.

[23] Kozakowska M., Pietraszek-Gremplewicz K., Jozkowicz A., Dulak J. (2015). The role of oxidative stress in skeletal muscle injury and regeneration: focus on antioxidant enzymes. *Journal of Muscle Research and Cell Motility*.

[24] Ragusa R. J., Chow C. K., Porter J. D. (1997). Oxidative stress as a potential pathogenic mechanism in an animal model of Duchenne muscular dystrophy. *Neuromuscular Disorders*.

[25] Matsumura C. Y., Menezes de Oliveira B., Durbeej M., Marques M. J. (2013). Isobaric tagging-based quantification for proteomic analysis: a comparative study of spared and affected muscles from *mdx* mice at the early phase of dystrophy. *PLoS One*.

[26] Carraro U., Coletti D., Kern H. (2014). The Ejtm specials “the long-term denervated muscle”. *European Journal of Translational Myology*.

[27] Powers S. K., Smuder A. J., Judge A. R. (2012). Oxidative stress and disuse muscle atrophy: cause or consequence?. *Current Opinion in Clinical Nutrition and Metabolic Care*.

[28] Pigna E., Greco E., Morozzi G. (2017). Denervation does not induce muscle atrophy through oxidative stress. *European Journal of Translational Myology*.

[29] Abruzzo P. M., di Tullio S., Marchionni C. (2010). Oxidative stress in the denervated muscle. *Free Radical Research*.

[30] Singla D. K., Singla R. D., McDonald D. E. (2008). Factors released from embryonic stem cells inhibit apoptosis in H9c2 cells through PI3K/Akt but not ERK pathway. *American Journal of Physiology-Heart and Circulatory Physiology*.

[31] Matsuda R., Nishikawa A., Tanaka H. (1995). Visualization of dystrophic muscle fibers in mdx mouse by vital staining with Evans blue: evidence of apoptosis in dystrophin-deficient muscle. *Journal of Biochemistry*.

[32] Sandri M., Carraro U. (1999). Apoptosis of skeletal muscles during development and disease. *The International Journal of Biochemistry & Cell Biology*.

[33] Tidball J. G., Albrecht D. E., Lokensgard B. E., Spencer M. J. (1995). Apoptosis precedes necrosis of dystrophin-deficient muscle. *Journal of Cell Science*.

[34] Carraro U., Franceschi C. (1997). Apoptosis of skeletal and cardiac muscles and physical exercise. *Aging*.

[35] Behrens L., Bender A., Johnson M. A., Hohlfeld R. (1997). Cytotoxic mechanisms in inflammatory myopathies. Co-expression of Fas and protective Bcl-2 in muscle fibres and inflammatory cells. *Brain*.

[36] Roy N., Mahadevan M. S., McLean M. (1995). The gene for neuronal apoptosis inhibitory protein is partially deleted in individuals with spinal muscular atrophy. *Cell*.

[37] Carotenuto F., Coletti D., di Nardo P., Teodori L. (2016). *α*-Linolenic acid reduces TNF-induced apoptosis in C2C12 myoblasts by regulating expression of apoptotic proteins. *European Journal of Translational Myology*.

[38] Bodine S. C., Stitt T. N., Gonzalez M. (2001). Akt/mTOR pathway is a crucial regulator of skeletal muscle hypertrophy and can prevent muscle atrophy in vivo. *Nature Cell Biology*.

[39] Gurpur P. B., Liu J., Burkin D. J., Kaufman S. J. (2009). Valproic acid activates the PI3K/Akt/mTOR pathway in muscle and ameliorates pathology in a mouse model of Duchenne muscular dystrophy. *The American Journal of Pathology*.

[40] Wang D. F., Yang H. J., Gu J. Q. (2015). Suppression of phosphatase and tensin homolog protects insulin-resistant cells from apoptosis. *Molecular Medicine Reports*.

[41] Grumolato L., Liu G., Mong P. (2010). Canonical and noncanonical Wnts use a common mechanism to activate completely unrelated coreceptors. *Genes & Development*.

[42] Nusse R. (2008). Wnt signaling and stem cell control. *Cell Research*.

[43] Brack A. S., Conboy M. J., Roy S. (2007). Increased Wnt signaling during aging alters muscle stem cell fate and increases fibrosis. *Science*.

[44] von Maltzahn J., Chang N. C., Bentzinger C. F., Rudnicki M. A. (2012). Wnt signaling in myogenesis. *Trends in Cell Biology*.

[45] Ikeya M., Takada S. (1998). Wnt signaling from the dorsal neural tube is required for the formation of the medial dermomyotome. *Development*.

[46] Kongkham P. N., Northcott P. A., Croul S. E., Smith C. A., Taylor M. D., Rutka J. T. (2010). The SFRP family of WNT inhibitors function as novel tumor suppressor genes epigenetically silenced in medulloblastoma. *Oncogene*.

[47] Coletti D., Daou N., Hassani M., Li Z., Parlakian A. (2016). Serum response factor in muscle tissues: from development to ageing. *European Journal of Translational Myology*.

[48] Hiroux C., Vandoorne T., Koppo K., de Smet S., Hespel P., Berardi E. (2016). Physical activity counteracts tumor cell growth in colon carcinoma C26-injected muscles: an interim report. *European Journal of Translational Myology*.

[49] Zampieri S., Doria A., Adami N. (2010). Subclinical myopathy in patients affected with newly diagnosed colorectal cancer at clinical onset of disease: evidence from skeletal muscle biopsies. *Neurological Research*.

[50] Berardi E., Aulino P., Murfuni I. (2008). Skeletal muscle is enriched in hematopoietic stem cells and not inflammatory cells in cachectic mice. *Neurological Research*.

[51] Yu A. P., Pei X. M., Sin T. K. (2014). Acylated and unacylated ghrelin inhibit doxorubicin-induced apoptosis in skeletal muscle. *Acta Physiologica*.

[52] von Maltzahn J., Bentzinger C. F., Rudnicki M. A. (2012). Wnt7a–Fzd7 signalling directly activates the Akt/mTOR anabolic growth pathway in skeletal muscle. *Nature Cell Biology*.

